# Elimination of epicardial late potentials by endocardial ablation in arrhythmogenic right ventricular cardiomyopathy

**DOI:** 10.1093/europace/euag231

**Published:** 2026-08-28

**Authors:** Alexis Hermida, Chiara Pavone, Romain Sauve, Géraldine Vedrenne, Denis Amet, Matthieu Gras, Bruno Degand, Cyrus Moini, Guillaume Duthoit, Mikael Laredo, Nicolas Badenco, François Lesaffre, Antoine Lepillier, Françoise Hidden Lucet, Estelle Gandjbakhch, Xavier Waintraub

**Affiliations:** Cardiac Arrhythmia Service, Amiens-Picardie University Hospital, 1 Rond-Point du Pr Christian Cabrol, F-80054 Amiens Cedex 1, France; Rhythmology Department, AP-HP, Hospital Pitie-Salpetriere, Institute of Cardiology, Paris, France; Biosense Webster, Paris, France; CNMR Maladies Cardiaques Héréditaires Rares, APHP, Hôpital Bichat Claude-Bernard, Paris, France; Cardiology Department, European Hospital Georges Pompidou, Paris, France; Cardiology Department, Nouvelle Clinique de Tours, Saint-Cyr-sur-Loire, France; Cardiology Department, La Miletrie University Hospital Centre, Poitiers, France; Cardiology Department, Jacques Cartier Private Hospital, Massy, France; Rhythmology Department, AP-HP, Hospital Pitie-Salpetriere, Institute of Cardiology, Paris, France; Rhythmology Department, AP-HP, Hospital Pitie-Salpetriere, Institute of Cardiology, Paris, France; Rhythmology Department, AP-HP, Hospital Pitie-Salpetriere, Institute of Cardiology, Paris, France; Cardiology Department, Hospital Robert Debré, Reims University Hospital, Reims, France; Cardiology Department, Centre Cardiologique du Nord, Saint-Denis, France; Rhythmology Department, AP-HP, Hospital Pitie-Salpetriere, Institute of Cardiology, Paris, France; Rhythmology Department, AP-HP, Hospital Pitie-Salpetriere, Institute of Cardiology, Paris, France; Rhythmology Department, AP-HP, Hospital Pitie-Salpetriere, Institute of Cardiology, Paris, France

**Keywords:** Arrhythmogenic right ventricular cardiomyopathy, Ventricular tachycardia, Catheter ablation, Epicardial substrate, Late potentials, Ablation index

Catheter ablation is frequently required for the treatment of ventricular tachycardia (VT) in patients with arrhythmogenic right ventricular cardiomyopathy (ARVC). Ablation strategies targeting noninducibility (NI) and late-potential (LP) abolition have been shown to be effective.^1^ Simultaneous endocardial and epicardial mapping in ARVC has demonstrated predominant epicardial substrate involvement in most VT cases.^2^ However, epicardial fat and proximity to coronary arteries may limit the feasibility or effectiveness of epicardial ablation.

We sought to (i) evaluate an endocardial-only ablation strategy guided by epicardial LP recordings to achieve LP elimination and NI and (ii) measure ablation index (AI) values associated with epicardial LP suppression by endocardial ablation, used as a surrogate for transmural lesion formation.

From 2019 onwards, this strategy was evaluated in consecutive ARVC patients referred for ventricular arrhythmia ablation. Endocardial and epicardial^3^ voltage mapping of the right ventricle (RV) was performed in sinus rhythm using bipolar thresholds of 0.5–1.5 mV for endocardial scar and 0.5–1.0 mV for epicardial scar, as previously defined.^4^ Late potentials were manually annotated. Programmed ventricular stimulation was performed down to S4. Whenever VT was inducible and haemodynamically tolerated, activation mapping was performed to identify the critical isthmus and exit site. Otherwise, substrate mapping combined with pace mapping was used to identify and validate the critical isthmus. Endocardial ablation was performed using an irrigated-tip catheter (Thermocool SF™, 35–40 W, 43°C) positioned opposite the area where epicardial LPs were recorded with a multielectrode diagnostic catheter (PentaRay™), aiming to eliminate the corresponding epicardial LPs. Each endocardial radiofrequency application was delivered under continuous epicardial LP monitoring, and the corresponding AI was recorded. Median AI values were subsequently calculated for each right ventricular region. Remapping confirmed LP abolition, and NI was reassessed. Follow-up relied on implantable cardioverter-defibrillator (ICD) telemonitoring or Holter/exercise testing. Patients were derived from the CARE registry, which was approved by the institutional ethics committee (CER-2023-PENE-CARE). All patients provided written informed consent.

Eleven patients were included [nine men, two women; median age 44 (34–51) years], including nine referred for recurrent VT (three redo procedures after prior endocardial-only ablation) and two referred for frequent premature ventricular contractions (Figure 1). The median number of ICD therapies before ablation was 4.5 (1.75–5.5) per patient. The clinical VT exit site was located in the RV outflow tract in five patients, the peritricuspid region in two, and the RV free wall in four. Substrate extent was greater in the epicardium than in the endocardium, with epicardial LP and scar areas of 39 (29–51) cm^2^ and 130 (95–136) cm^2^, respectively, compared with 19 (11–32) cm^2^ (*P* = 0.004) and 24 (5–36) cm^2^ (*P* = 0.001) endocardially (Figure 1). One patient required complementary epicardial ablation to achieve complete LP elimination. The median ablation time was 48 (43–56) min. Late-potential elimination was achieved in all patients. Ventricular tachycardia was noninducible in all tested patients; one patient was not tested because of haemodynamic instability. One patient developed retrosternal haematoma with spontaneous resolution. The median AI values of endocardial radiofrequency applications delivered to eliminate epicardial LPs were 604 (528–679) for the inferior wall, 546 (409–694) for the RV outflow tract, 577 (401–692) for the peritricuspid region, and 603 (510–714) for the RV free wall. During a median follow-up of 17 (13–52) months, VT recurred in three patients.

**Figure 1:**
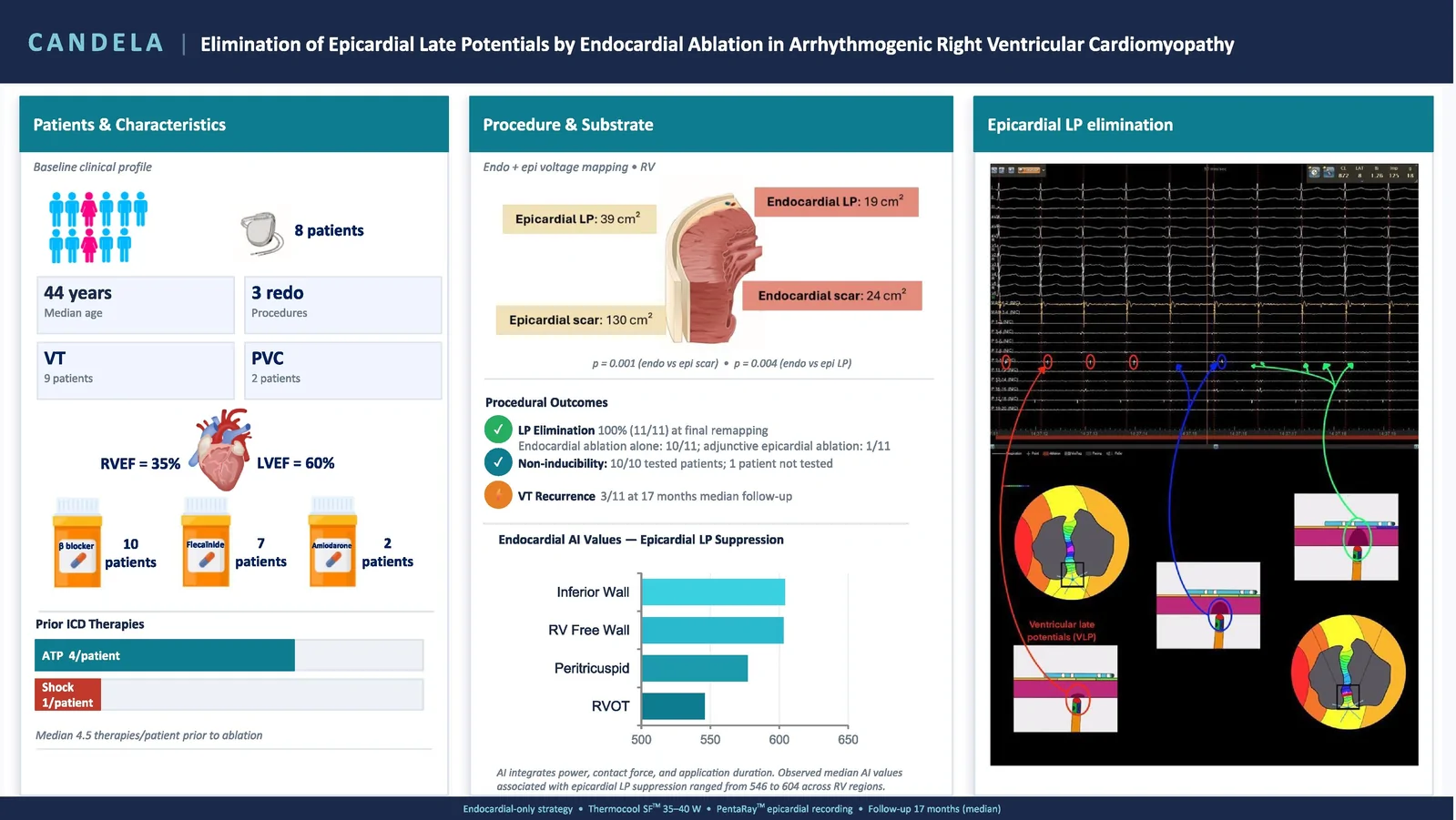
Left: clinical characteristics of the included patients. Center: Procedural characteristics, including epicardial and endocardial scar and late-potential extent, and endocardial ablation index values associated with epicardial LP suppression according to the targeted right ventricular region. Right: Representative example of epicardial late-potential elimination, recorded with a multielectrode mapping catheter (PentaRay™, Biosense Webster), achieved by endocardial ablation. Abbreviations: AI = ablation index; ATP = antitachycardia pacing; ICD = implantable cardioverter-defibrillator; LP = late potential; LVEF = left ventricular ejection fraction; PVC = premature ventricular contraction; RV = right ventricle; RVEF = right ventricular ejection fraction; RVOT = right ventricular outflow tract; VT = ventricular tachycardia.

In this case series, an endocardial ablation strategy guided by epicardial recordings achieved complete LP elimination without complementary epicardial ablation in 10 of 11 patients with ARVC, and VT noninducibility in all 10 tested patients, with a low recurrence rate at mid-term follow-up.

Epicardial substrate involvement is a hallmark of ARVC-related VT.^2^ Although epicardial ablation is often required, it may be challenging or incomplete in clinical practice, particularly in the presence of epicardial fat or when critical structures limit energy delivery. In selected cases, incomplete LP elimination may occur, supporting the use of alternative approaches. In addition, repeated epicardial access may be limited by pericardial adhesions. In this context, while epicardial substrate modification may be achieved by endocardial ablation, a key challenge remains the noninvasive identification of epicardial substrate, which would be required to enable a fully endocardial strategy. Despite a greater epicardial substrate burden, only one patient required complementary epicardial ablation to achieve complete epicardial LP elimination, suggesting that an endocardial-only approach may be feasible in selected ARVC patients.

Consistently, our findings suggest that endocardial ablation delivered opposite epicardial LPs may result in elimination of the corresponding epicardial signals. Epicardial LP elimination may therefore serve as a pragmatic electrophysiological surrogate for sufficient lesion depth, without directly assuming histological transmurality.

An original contribution of this study is the reporting of endocardial AI values associated with epicardial LP suppression. Median AI values ranged approximately from 550 to 600 depending on the right ventricular region. Although AI does not directly measure lesion depth, it integrates power, contact force, and application duration and has been associated with lesion size and durability in prior studies.^5^ These values may serve as practical benchmarks to achieve sufficient lesion depth in the RV when targeting epicardial LPs from the endocardium.

This study is limited by its retrospective design and small sample size. Transmurality was inferred using electrophysiological surrogates rather than direct imaging or histological confirmation, VT morphology and cycle length at recurrence were not consistently available, and follow-up duration was limited. Accordingly, these findings should be considered hypothesis generating and warrant confirmation in larger prospective studies.

## Data Availability

The data underlying this article will be shared on reasonable request to the corresponding author.
